# The Use of Different Cell Wall Degrading Enzymes for Pectin Extraction from Carrot Pomace, in Comparison to and in Combination with an Acid Extraction

**DOI:** 10.3390/foods14030435

**Published:** 2025-01-28

**Authors:** Elien De Laet, Tom Bernaerts, Lise Morren, Hanne Vanmarcke, Ann M. Van Loey

**Affiliations:** Laboratory of Food Technology, Department of Microbial and Molecular Systems, KU Leuven, Kasteelpark Arenberg 22, B-3001 Leuven, Belgium

**Keywords:** pectin, acid extraction, enzyme-assisted extraction, combined extraction processes on carrot pomace, molecular pectin structure

## Abstract

The effect of different cell wall degrading enzymes, cellulase (C) and hemicellulase (HC), during the enzyme-assisted extraction (EAE) of pectin from carrot pomace was investigated. The EAE with C and a heat treatment resulted in a pectin yield, purity, and molecular structure comparable to an acid extraction (AE), except for a slightly lower molar mass and a slightly higher degree of methylesterification. The addition of HC had a negligible influence on the pectin yield and structure and mainly resulted in more hemicellulose co-extraction. Overall, the AE still resulted in the highest pectin yield, but, despite the much milder extraction conditions, the optimal EAE process resulted in 80% of the pectin yield of the AE. Additionally, this study investigated an EAE with C in combination with an AE, and both combination treatments, i.e., EAE as pretreatment or as an additional treatment, resulted in a significant increase in the pectin yield (up to 72%), while minor structural differences were observed in the extracted pectin. Overall, it can be concluded that the EAE process can be used as a more environmentally friendly alternative for the AE or that EAE can be used in combination with an AE to improve the efficiency of the extraction process.

## 1. Introduction

In recent years, the interest in pectin as a functional (food) ingredient has strongly increased because of the broad range of potential applications of pectin in several industries [1,2,3]. Pectin owes this versatility to its complex molecular structure, as pectin has, by far, the most complex structure of all cell wall polysaccharides [4,5]. Within the pectin polysaccharide structure, three main structural domains can be distinguished: homogalacturonan (HG), rhamnogalacturonan I (RG I), and rhamnogalacturonan II (RG II) [6]. HG is the most abundant pectin structural domain and is a homopolymer of α-(1,4)-linked galacturonic acid (GalA) residues. A proportion of the GalA residue can be methyl esterified at the C6-position, and this percentage is indicated by the degree of methylesterification (DM), and/or can carry an acetyl group at the O2- and/or O3-position. Due to its linear nature, this domain is often referred to as the ‘smooth’ region of the pectin structure [2,4,7]. RG I, on the contrary, is composed of several repeats of the disaccharide [2)-α-L-Rha-(1,4)-α-D-GalA-(1], and side chains of arabinan and (arabino)galactan can be attached to 20–80% of the rhamnose (Rha) units, resulting in a much more branched pectin domain, often referred to as the ‘hairy’ region of the pectin structure [2,8,9]. RG II is, by far, the most complex and preserved structural domain and is composed of a linear HG backbone to which four different hetero-oligomeric side chains, containing up to eleven different sugar residues, can be attached [10,11,12]. Besides these three common pectin domains, a substituted xylogalacturonan can also be found to a smaller extent [13].

In vivo, pectin is present in the complex three-dimensional structure of the cell wall of dicotyl plants, where it is present in the middle lamella and the primary cell wall [2]. Therefore, an extraction process is crucial to liberate the pectin polysaccharides from the cell wall matrix. On an industrial scale, this extraction process is nowadays mainly performed as an acid extraction from citrus peel and apple pomace, which includes an extraction process at a low pH (1.5–3), using strong mineral acids, and high temperatures (50–100 °C), and these extraction processes are performed for up to several hours under continuous stirring [5,9]. Because of the prolonged extraction time and the high extraction temperatures, this conventional extraction technique is accompanied by high energy consumption, and, in combination with the large amount of acidic waste water produced, this technique has a large environmental impact [2,9]. Therefore, in recent years, the potential of enzyme-assisted extraction (EAE) was investigated, on the one hand, as a more environmentally friendly alternative for acid extraction, and, on the other hand, to potentially improve the efficiency of the acid extraction [14,15,16]. For this EAE, it can either be chosen to use cell wall degrading enzymes without or with a very low pectinolytic activity to extract relatively intact pectin polymers or use pectinolytic enzymes that degrade the pectin structure and extract shorter pectin fragments. As in this study, the aim was to maintain the pectin structure and, therefore, the functional properties as much as possible. The latter approach was not desirable, and the EAE process with enzymes with no or limited pectinolytic activity was selected [15,17].

In the literature, a wide range of multicatalytic enzyme preparations and (single) enzymes were used for the EAE of pectin from different plant-based side streams as potential pectin sources [14,17,18,19,20,21,22]. However, it should be noted that varying results were obtained using the different multicatalytic enzyme preparations, as some studies report a higher extraction yield in comparison to the acid extraction [19,21,23], whereas other studies report a lower extraction yield [21,24]. Additionally, no consensus was reached regarding the relative importance of cellulase and hemicellulase activity during the EAE process. In the studies of Abou-Elseoud et al. (2021), Idrovo Encalada et al. (2019), Milošević & Antov (2022), Naghshineh et al. (2013), and Shkodina et al. (1998) [14,16,18,25,26], it was reported that hydrolysis of the cell wall network and the resulting pectin extraction was more efficient with cellulase than with hemicellulase, or, more specifically, xylanase, for sugar beet pulp, carrot tissue, butternut squash, lime peel, and pumpkin tissue. Wikiera et al. (2016) [22] reported the exact opposite for apple pomace.

In this study, the effect of different cell wall degrading enzymes, cellulase, hemicellulase, and a combination of both, during the EAE process from carrot pomace was evaluated to elaborate on the relative importance of both cell wall degrading enzymes. The results obtained in this study were compared with an acid extraction process analogous to the industrial pectin extraction process; as in previous studies, a broad range of extraction conditions (pH 1–2 and 70–100 °C) was used for this acid extraction, which influences the comparison with the EAE process. Besides the effect on the extraction yield on a mass basis, the current study also included the pectin extraction yield, a parameter that corrects for the potential co-extraction of compounds other than pectin, which is not the case for the extraction yield on a mass basis (mostly studied in the literature). The enzyme activities of both enzymes were also reported in this study, which was not always the case for the studies available in the literature. Additionally, the current study investigated, for the first time, whether the combination of the EAE with an acid extraction, either as pretreatment or as an additional treatment on the acid residue, can be used to increase the efficiency of the pectin extraction process from carrot pomace. This side stream, obtained in the carrot juice production process, was selected as biomass in the current study because of its high dietary fiber content [16] and because very large amounts of carrot pomace are being produced as the consumption of vegetable juice and the production of natural carrot-based colorants is increasing [27,28]. Idrovo Encalada et al. (2019) [16] already investigated the EAE of pectin from this biomass using cellulase and hemicellulase separately. However, the exact enzyme activities were not reported, and a comparison with an acid extraction was also not included in this study [16].

## 2. Material and Methods

The structure and content of this section are based on the previous work of De Laet et al. (2024) [29].

### 2.1. Material

For the production of the carrot pomace, whole carrots (*Daucus carota* L. cv. Nerac) were purchased from a fruit and vegetable store in Ghent and subjected to a pilot-scale juice production process. After removal of the ends of the carrots, two consecutive grinders (KWEM 1000, Kreuzmayr Maschinenbau GmbH, Wallem, Germany; Multicut 1500, Bruckner Liquid Food Tech GmbH, Abstatt, Germany) were used to crush the carrots, and on the obtained carrot mash a juice extraction was performed (Vaculiq 1000, Vaculiq GmbH & Co. KG, Hamminkeln, Germany). This juicing process resulted in a carrot juice and a carrot pomace, the side stream of the juice extraction, and this carrot pomace (CP) was the starting material in this study. In order to obtain a stable biomass, the pomace was blanched for 5 min at 100 °C and kept at −40 °C (below the glass transition temperature of carrot (approx. −32 °C)) [30].

### 2.2. Characterization and Pretreatment of the Starting Material

#### 2.2.1. Dry Matter Content

The dry matter content of the CP was determined in triplicate by drying in a vacuum oven (UniEquip 1445-2, Planegg, Germany) under a pressure of 0.2–0.8 bar and at a temperature of 70 °C for 4 h.

#### 2.2.2. Production of the Alcohol Insoluble Residue

The alcohol insoluble residue (AIR) was isolated to gain more insight into the amount and the molecular structure of the pectin initially present in the biomass. Therefore, the monosaccharide content (galacturonic acid and neutral sugars) (Section 2.5.1) in the AIR was determined. The method for the isolation of the AIR was based on the procedure of McFeeters & Armstrong (1984) [31]. Briefly, 30 g of wet carrot pomace suspended in 192 mL of technical ethanol (99%) was mixed three times for a duration of 6 s (B 400 mixer, Büchi, Flawil, Switzerland). After a vacuum filtration step (filter paper MN615 Ø 90 mm, Macherey-Nagel, Düren, Germany), the residue retained on the filter paper was resuspended in 96 mL technical ethanol and subjected to three mixing steps. The residue obtained after a second vacuum filtration step was suspended in 96 mL of technical acetone and stirred for a duration of 10 min. After a final vacuum filtration step, the residue retained by the filter was dried in an oven at 40 °C for 24 h, resulting in the AIR.

#### 2.2.3. Pretreatment of the Starting Material

To obtain a starting material with a standardized small particle size (compared to the wet pomace), the CP was subjected to a dry ball milling treatment. Therefore, the wet pomace was first lyophilized for a duration of 24 h (Alpha 2–4 LSC plus, Christ, Osterode, Germany). The lyophilized carrot pomace was subjected to a ball mill treatment (Ball Mill MM400, Retch GmbH, Haan, Germany) at 30 Hz for 30 s. Four balls made of steel with a diameter of 15 mm were used for this ball mill treatment. The ball-milled carrot pomace was stored in a desiccator until used for the extraction process described below.

### 2.3. Different Pectin Extraction Processes

#### 2.3.1. Acid Extraction

The acid extraction was performed in duplicate, analogous to the procedure of Willemsen et al. (2017) [32]. In short, 3 g of ball-milled carrot pomace (Section 2.2.3) was added to 200 mL of preheated (80 °C) demineralized water and stirred for 30 min at 80 °C. After pH adjustment (pH 1.6) using a 7 M HNO_3_ solution, the extraction process was carried out at 80 °C for a duration of 1 h. Afterward, an ice bath was used to cool the mixture and after a centrifugation treatment at 4000× *g* and 4 °C for a duration of 10 min (Avanti JXN-26, Beckman Coulter Inc., Indianapolis, IN, USA); vacuum filtration (MN615 Ø 90 mm, Macherey-Nagel, Düren, Germany) was performed to obtain the fraction rich in pectin as the filtrate, while the acid residue (AR) was retained by the filter. Alcohol precipitation was performed on the pectin-rich fraction to precipitate the extracted pectin (Section 2.3.4).

#### 2.3.2. Enzyme-Assisted Extraction

**Figure 1:**
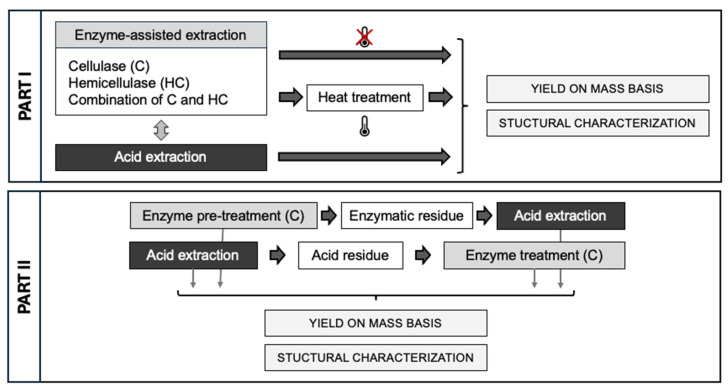
Schematic representation of the different extraction processes.

For the enzyme-assisted extraction (EAE) processes (all performed in duplicate), two different enzymes and a combination of these two enzymes were used, cellulase from *Trichoderma reesei* with an enzyme activity of 756 β-glucanase units (EGU)/g (one unit will liberate 1 µmol of D-glucose from cellulose per hour at pH 5 and 37 °C) and hemicellulase from *Aspergillus niger* with a hemicellulase activity of 1500 U/g (one unit will produce a relative fluidity change of 1 per 5 min using locust bean gum as a substrate at pH 4 and 40 °C) and a negligible cellulase activity (20 U/g). This hemicellulolytic mixture contained xylanase and mannanase activities, among others. For all EAE processes, 3 g of ball-milled carrot pomace (Section 2.2.3) was added to 200 mL of sodium acetate buffer (100 mM) at pH 5. The samples were placed in a shaking water bath and preheated to 50 °C, after which the different enzymes (cellulase (C), hemicellulase (HC), and cellulase + hemicellulase (C + HC)), were added in an enzyme concentration of 100 U/g dry biomass for each enzyme, and the extraction process was performed for 24 h at 50 °C. For the three different processes (C, HC, and C + HC), the enzyme treatments were either followed by no heat treatment or a heat treatment of 5 min at 80 °C. For the samples without heat treatment, the pectin-rich filtrate was separated from the enzymatic residue (ER) by vacuum filtration (MN615 Ø 90 mm, Macherey-Nagel, Düren, Germany) and, after pH adjustment to pH 2 (with 0.1 and 1 M HCl), an alcohol precipitation was performed on the pectin-rich fraction (Section 2.3.4). For the samples with the heat treatment, the pH was adjusted to pH 4 (with 0.1 and 1 M HCl) (to reduce β-elimination), and a heat treatment of 5 min at 80 °C was performed under continuous stirring. Afterward, the same steps were followed as for the samples without heat treatment. Additionally, a control extraction was performed completely analogously to the EAE processes, but without the addition of enzymes (Figure 1).

#### 2.3.3. Combination Treatments

**Figure 2:**
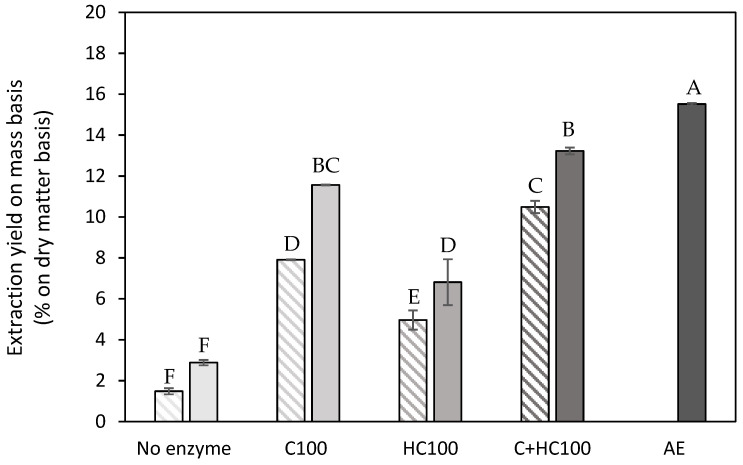
Extraction yield on a mass basis (% on dry matter basis) obtained after a control extraction (no enzyme) and the different enzyme-assisted extractions (cellulase (C), hemicellulase (HC), combination (C + HC)) without (shaded) and with (full) heat treatment of 5 min at 80 °C, in comparison to an acid extraction (AE) (dark grey). 100: concentration of 100 U/g for each enzyme. The error bars represent the standard deviation. A Tukey HSD test with *p* < 0.05 was used to evaluate whether differences in extraction yield on a mass basis were significant (different letters).

The enzyme-assisted extraction (EAE) with cellulase (C) in a concentration of 100 U/g dry biomass was combined with an acid extraction (AE), either as an additional extraction process on the acid residue or as a pretreatment (Figure 2). In the first option (AE→EAE), the AE was performed analogously to in Section 2.3.1, and the acid residue (AR) was freeze-dried (Alpha 2–4 LSC plus, Christ, Osterode, Germany) and ball milled (using the same condition as in Section 2.2.3). The consecutive EAE process was performed analogously to in Section 2.3.2 on a smaller scale, using 1.5 g of ball-milled AR. In the second option (EAE→AE), the EAE process was performed as described in Section 2.3.2, with and without heat treatment, and the enzymatic residue (ER) was lyophilized for a duration of 24 h. Next, an acid extraction (analogously to Section 2.3.1) was performed on a smaller scale, using 1.5 g of the lyophilized ER. In both combination processes, the different extraction steps were separated, and the pectin-rich filtrate obtained in the different extraction steps was subjected to an alcohol precipitation (Section 2.3.4).

#### 2.3.4. Alcohol Precipitation

The alcohol precipitation of the pectin-rich fractions was performed as described by De Laet et al. (2024) [29]. The fraction rich in pectin obtained after vacuum filtration (volume V) was added to four volumes of technical ethanol (99%), which resulted in a mixture with a final ethanol concentration of 80%. After stirring for a duration of 10 min, the mixture was allowed to stand for 1 h at ambient temperature. The precipitated material was retained using vacuum filtration and washed with technical ethanol (same volume V as the original pectin-rich fraction). After this washing step, the precipitate was redissolved in 50 mL of demineralized water. After pH adjustment to a pH of 6.5, the dissolved pectin fraction was lyophilized for a duration of 24 h and kept in a desiccator.

### 2.4. Determination of the Extraction Yield on Mass Basis

The following equation was used to determine the extraction yield on a mass basis:

$$\;\;\;\;\;\;\;\;\;Extraction\;yield\;\left(\%\right)=\frac{g\;extract\;(after\;freeze\;drying)}{g\;carrot\;pomace\;(on\;dry\;matter\;basis)}\times 100$$

### 2.5. Determination of the Molecular Structure of the Extracted Materials

#### 2.5.1. Monosaccharide Content

On the one hand, the monosaccharide content was measured in the AIR to determine the amount of pectin initially present in the biomass. On the other hand, the monosaccharide content of the different extracted materials was measured to investigate the efficiency of the extraction processes (pectin extraction yield) and the molecular structure of the extracted material.

The determination of the GalA content consisted of hydrolysis with concentrated sulphuric acid (95%) [33] and a spectrophotometric analysis based on the procedure of Blumenkrantz & Asboe-Hansen (1973) [34]. After performing the hydrolysis in duplicate, a sulphuric acid with sodium tetraborate solution (0.0125 M) was added to 0.6 mL of the hydrolyzed sample and incubated in an oil bath at 100 °C for 5 min. After cooling, 60 µL of 0.15% (*w*/*v*) *m*-hydroxydiphenyl in 0.5% NaOH was added, and, after mixing for 1 min, the absorbance at 520 nm was measured spectrophotometrically (Spectrophotometer Genesys 30 Vis, Thermo Fisher, Waltham, MA, USA). For each hydrolysate, the absorbance was measured in triplicate, and a blank was measured by adding 0.5% NaOH.

The neutral monosaccharide profile was determined through high-performance anion-exchange chromatography with pulsed amperometric detection using a Dionex ICS-6000 system with an ICS-6000 pump and an ED50 chemical detector (Dionex, Sunnyvale, CA, USA). To obtain the different monosaccharides coming from pectin and hemicellulose, matrix hydrolysis was performed. Therefore, the extracted polymers were subjected to a hydrolysis step in a 4% H_2_SO_4_ solution, as described by Yeats et al. (2016) [35]. Overnight, 2 mg of extracted material (or AIR) was dissolved in 2.8 mL ultrapure water, and 100 µL 72% H_2_SO_4_ was added to obtain a final H_2_S0_4_ concentration of 4%. The Saeman hydrolysis, on the other hand, was performed by first incubating the samples in a 72% H_2_SO_4_ solution (without the overnight dissolving step) to determine the glucose content originating from both cellulose and hemicellulose [35]. All mixtures were incubated for 1 h at 121 °C, and, after cooling, 2.32 mL of 1 M NaOH was added to neutralize the acid. Lastly, the neutralized mixture was filtered with a 0.45 µm syringe filter (Chromafil^®^ A-45/25, Macherey-Nagel, Düren, Germany). The HPLC method for separation and quantification of the monosaccharides was based on the procedures described by Yeats et al. (2016) [35]. A CarboPac™ PA20 guard column (30 × 3 mm) followed by a CarboPac™ PA20 column (150 × 3 mm) was used. For the separation of fucose (Fuc), galactose (Gal), glucose (Glc), xylose (Xyl), and mannose (Man), 2 mM NaOH was selected, while an elution solution of 18 mM of NaOH was used for the separation of rhamnose (Rha) and arabinose (Ara). To quantify the different monosaccharides, external calibration curves were obtained from monosaccharide standards (0.05–5 ppm), and a correction factor was calculated to correct for possible degradation of the neutral sugars during the hydrolysis protocol.

#### 2.5.2. Protein Content

The Dumas method was performed to determine the protein content of the different samples in duplicate, as described by Jung et al. (2003) [36], with some adjustments. Briefly, this procedure determines the total nitrogen content by incinerating tin weighing cups with 2 mg of sample in an EA 1108 CHNS-O elemental analyzer (CE Instruments, Thermo Fisher Scientific, Waltham, MA, USA) at 1020 °C. A factor of 6.25 was used to convert the measured nitrogen content into an estimation of the protein content.

#### 2.5.3. Degree of Methylesterification

Fourier transform infrared (FTIR) spectroscopy (FTIR-8400S, Shimadzu, Kyoto, Japan), as described by Kyomugasho et al. (2015) [37], was used to determine the DM of the different extracted pectins in duplicate. In this procedure, IR spectra were measured and the intensities of the peaks at 1600 cm^−1^ (carboxyl groups COO^−^) and 1740 cm^−1^ (carbonyl groups C=O) were converted into the ratio of the amount of carboxyl groups that carries a methyl ester (1740 cm^−1^) to the total amount of carboxyl groups (sum of both peaks). Finally, the calibration curve obtained by Kyomugasho et al. (2015) [37] was used to convert this ratio into a DM value.

#### 2.5.4. Molar Mass Distribution

Based on the procedure of Shpigelman et al. (2014) [38], high-performance size exclusion chromatography with a multiangle laser light scattering (MALLS) detector (PN3621, Postnova analytics, Landsberg am Lech, Germany), diode array detector (G1316A, Agilent Technologies, Diegem, Belgium), and refractive index (RI) detector (Shodex RI-101, Showa Denko K.K., Kawazaki, Japan) was performed to determine the molar mass distribution of the different extracts. Overnight, the extracted materials were dissolved in an acetic acid buffer (0.1 M) with NaNO_3_ (0.1 M) at a pH of 4.4 (2% solution) and, before injection, the mixture was filtered (Chromafil^®^ A-45/25, Macherey-Nagel, Düren, Germany). The different polymers (in 100 µL) were separated over three different Waters columns in series (Ultrahydrolgel 250 (8 × 10^4^ g/mol), 1000 (4 × 10^6^ g/mol), and 2000 (1 × 10^7^ g/mol), with an acetic acid buffer (pH 4.4) as an elution solvent over a period of 120 min. The concentration profile was obtained based on the RI signal and a d*n*/d*c* value of 0.146 mL/g, and the different molar masses were calculated with the Debye fitting method performed on the MALLS signal.

### 2.6. Calculations

To correct for the possible co-extraction of compounds other than pectin, the parameter pectin extraction yield was defined and determined. This parameter considers the extraction yield (only determined on a mass basis) (Section 2.4) as well as the purity of the extract. This purity is calculated by taking the sum of the different pectin-related monosaccharides (GalA, Gal, Ara, Rha, Xyl, and Fuc) in 100 g of extracted material. The pectin extraction yield was calculated as the ratio of the amount of pectin extracted to the total amount of pectin initially present in the biomass, and was calculated as described by De Laet et al. (2024) [29] using the following equation:

$$Pectin\;extraction\;yield\;\left(\%\right)=\frac{\frac{g\;extracted\;material}{g\;carrot\;pomace}\times \frac{g\;pectin}{100\;g\;extracted\;material}}{\frac{g\;AIR}{g\;carrot\;pomace\;}\times \frac{g\;pectin}{100\;g\;AIR}}\times 100$$

in which the amount of pectin was calculated as the sum of all pectin-related monosaccharides.

Analogous to the work of Denman & Morris (2015) [39], different sugar ratios were calculated with minor modifications to gain more insight into the molecular structure of the extracted material. For these different calculations, the monosaccharide contents were converted into molar concentrations. The contributions of the HG and RG I domains to the extracted material were calculated as %HG=GalA−Rha and %RG I=2Rha+Ara+Gal, respectively, and the length of the RG I side chains and the relative contributions of arabinose and galactose to these side chains were calculated as (Ara+Gal)/Rha and Gal/Ara, respectively.

### 2.7. Statistical Analysis

Production of the different samples under investigation in the current study was performed in duplicate, and the different analyses were carried out in duplicate as well. Therefore, the data shown in this study represent the average of four data points with a standard deviation. Error propagation was used for the calculation of the pectin extraction yield, the pectin purity, and the different monosaccharide ratios, and the 95% confidence intervals were used to evaluate whether the differences between the different samples were significant. For protein content, DM, and average molar mass, Tukey HSD (Honest Significant Difference) tests (*p* < 0.05) were used, which were performed using statistical software (JMP Pro 17, Cary, NC, USA).

## 3. Results and Discussion

Current research can be subdivided into two parts. In the first part, the effect of different cell wall degrading enzymes, cellulase, hemicellulose, and a combination of both enzymes and the impact of the addition of heat treatment during the enzyme-assisted extraction (EAE) were investigated. In the second part, the most promising enzyme-assisted extraction process was combined with an acid extraction, either as an additional extraction step on the acid residue or as enzymatic pretreatment before the acid extraction.

### 3.1. Effect of Different Cell Wall Degrading Enzymes and a Heat Treatment

Initially, the effect of two different cell wall degrading enzymes, cellulase (C) and hemicellulase (HC), and the combination of these two enzymes (C + HC) during the EAE process was investigated. The research hypothesis that the EAE treatment might result in the alteration of the surroundings of the pectin in the cell wall but that an additional heat treatment at an increased temperature might be needed to solubilize the pectin, resulting in the addition of a heat treatment after the enzymatic treatment. Therefore, the EAE processes were evaluated without and with consecutive heat treatment for all enzyme(s) (combinations). The heat treatment was performed at 80 °C, the same temperature as was used for the acid extraction of pectin, and a treatment time of 5 min was selected, based on preliminary experiments, which showed no further influence on the extraction yield on a mass basis for prolonged heating times.

#### 3.1.1. Extraction Yield

**Figure 3:**
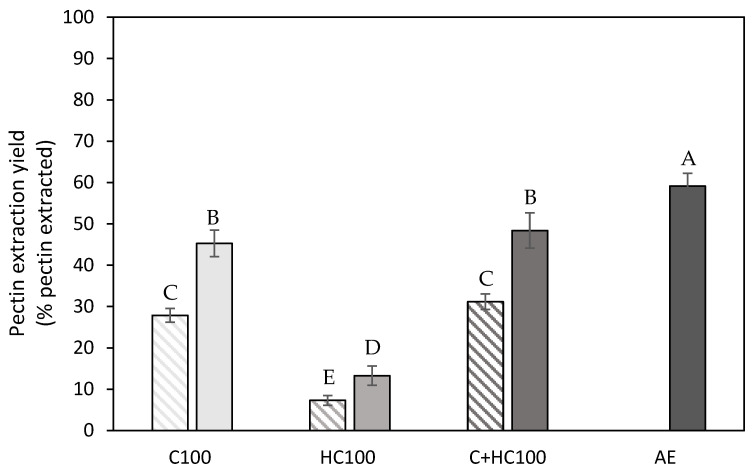
Pectin extraction yield (% pectin extracted) obtained after the different enzyme-assisted extractions (cellulase (C), hemicellulase (HC), combination (C + HC)) without (shaded) and with (full) heat treatment of 5 min at 80 °C, in comparison to an acid extraction (AE) (dark grey). 100: concentration of 100 U/g for each enzyme. The error bars represent the standard deviation. A 95% confidence interval was used to evaluate whether differences in pectin extraction yield were significant (different letters).

In Figure 2, the extraction yield (mass basis) is shown for all EAE processes, without and with a heat treatment, as well as for the control extraction (same conditions without the addition of enzyme(s)) and for the acid extraction. As expected, the lowest extraction yields on a mass basis were observed for the control extractions, as, under the mild extraction conditions used (pH 5 and 50 °C), only a small amount of material was extracted. When comparing the two cell wall degrading enzymes, the addition of cellulase (C) during the EAE process resulted in the largest increase in the extraction yield, and combining both enzymes resulted in a further increase in the extraction yield by approx. 2% on a dry matter basis. In the study of Idrovo Encalada et al. (2019) [16] on carrot tissue, the same trend was observed for the use of cellulase and hemicellulase, i.e., a significantly increased extraction yield with cellulase and a less pronounced effect with the addition of hemicellulase. The combined effect of both enzymes was not included in their study. A comparison of the absolute extraction yield values between both studies was not possible as the study of Idrovo Encalada et al. (2019) [16] only reported the amounts of enzyme added without reporting the actual enzyme activities. Additionally, it can also be observed (Figure 3) that, for all EAE processes, the addition of the heat treatment resulted in a significant increase in the extraction yield. The extraction yields for the EAE processes with C and C + HC were in the same order of magnitude (8–11%) as the extraction yield for an acid extraction performed on dried carrot pomace reported in the literature using comparable extraction conditions as used in the current study [40]. However, in the current study, a significantly higher extraction yield was achieved with the acid extraction, with an extraction yield on a mass basis of up to almost 16%. This higher extraction yield, compared to the study of Jafari et al. (2017) [40], can potentially be attributed to the ball mill pretreatment of all starting materials in the current study, as, in the previous work of De Laet et al. (2024) [29], it was already shown that the addition of a ball mill treatment resulted in a significantly increased extraction yield. In the present study, the ball mill pretreatment was included both before the EAE processes as well as prior to the AE to allow for a fair comparison. Overall, it can be observed that the EAE processes under investigation cannot be used to increase the extraction yield on a mass basis, although some studies in the literature suggested otherwise [17,23,25]. Naghshineh et al. (2013) [25] reported an increase in the extraction yield using an EAE with cellulase compared to an acid extraction on lime peel. However, it should be noted that, in this experimental approach, an additional boiling step of 5 min was implemented (to inactivate the enzymes), while the acid extraction was performed at a temperature of only 70 °C. Panouillé et al. (2006) [17] also reported an increase in extraction yield with EAE on chicory roots, using a combination of cellulase and protease. For pumpkin tissue, Ptichkina et al. (2008) [23] described an increase in extraction yield using an enzyme mixture, but this mixture also had a relatively high polygalacturonase activity, which will, of course, influence the extraction efficiency, yet at the cost of a reduced molar mass of the extracted pectin polymers. Also, the fact that different biomasses were used in the latter study might also have an influence. In the research performed on carrot tissue [16], comparison with an acid extraction (AE) was not included, and, therefore, for this specific biomass, comparison with the literature data was not feasible.

#### 3.1.2. Pectin Extraction Yield

As the extraction yield on a mass basis (Figure 2) also includes the potential co-extraction of compounds other than pectin, it does not allow us to distinguish between the pectin and co-extracted polymers. Therefore, a structural characterization was performed for all extracted materials (except for the control extractions) to gain insight into the amount of pectin extracted. Therefore, the pectin extraction yield was calculated as described in Section 2.6, where the pectin extraction yield was defined as the percentage of pectin that was extracted during the extraction process of the total amount of pectin initially present in the biomass. The initial pectin content was determined on the AIR and was found to be 2.35 ± 0.06 g pectin per 100 g of wet carrot pomace (CP) and the dry matter content of this wet carrot pomace amounted to 12.1 ± 0.2% [29].

In Figure 3, the pectin extraction yield is shown, and, in general, the same trends can be observed for the extraction yield on a mass basis. The addition of hemicellulase (HC) during the EAE process resulted in the lowest pectin extraction yield, whereas the addition of cellulase (C) resulted in a much higher efficiency. Additionally, it can be observed that the addition of hemicellulase in combination with cellulase (C + HC) did not result in an increased pectin extraction yield compared to the use of cellulase alone (C), as, for both extraction processes, a comparable pectin extraction yield was observed. This finding does confirm previous reports in the literature, mentioning that mainly the cellulose in the three-dimensional network of the primary cell wall should be degraded for the extraction of pectin (on a mass basis) [16]. A possible explanation for the higher efficiency with cellulase compared to hemicellulase might be the fact that cellulase enzymes not only degrade the cellulose polymers but also (part of) the xyloglucan polymers, which have a comparable backbone, to a more limited extent [41]. When comparing the extraction process with C to the combination of both enzymes, a higher extraction yield on a mass basis was obtained with the combination treatment, while a comparable pectin extraction yield was obtained for both processes. This indicates that the HC action during the combination treatment does not result in the extraction of pectin polymers, which will be confirmed later (Section 3.1.3). Additionally, the addition of heat treatment (5 min at 80 °C) resulted, for all treatments, in a significantly increased pectin extraction yield. As pectin solubilization is a temperature-driven process [42], the elevated temperatures during the heat treatment caused the solubilization of an additional amount of pectin, which was potentially more accessible due to the action of the enzymes. When comparing the different EAE processes to the AE, the highest extraction efficiency was still achieved with the acid extraction, which can most probably be explained by the much more severe extraction conditions used with the AE process (lower pH and higher temperature). However, it should be noted that the optimal EAE process (C + HC100 with heat treatment) resulted in a pectin extraction yield amounting to up to 80% of the pectin extraction yield achieved with the AE despite the much milder extraction conditions.

#### 3.1.3. Composition of the Extracted Material

**Figure 4:**
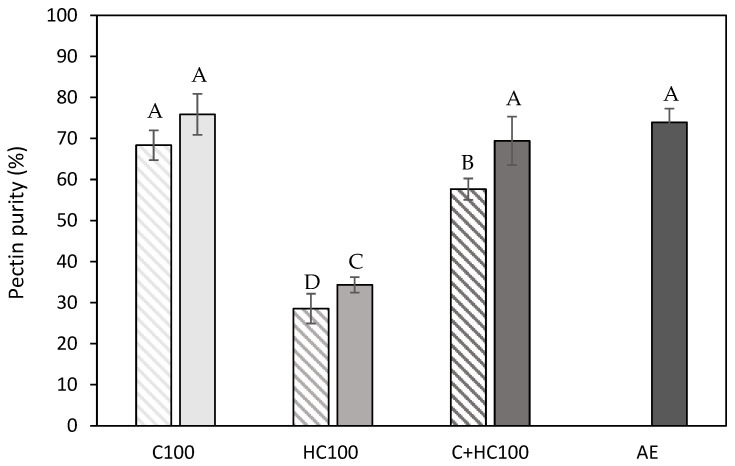
Pectin purity (%) of the extracted materials obtained after the different enzyme-assisted extractions (cellulase (C), hemicellulase (HC), combination (C + HC)) without (shaded) and with (full) heat treatment of 5 min at 80 °C, in comparison to an acid extraction (AE) (dark grey). 100: concentration of 100 U/g for each enzyme. The error bars represent the standard deviation. A 95% confidence interval was used to evaluate whether differences in pectin purity were significant (different letters).

To gain more insight into the composition of the extracted materials, the results obtained in the structural characterization were used to determine the pectin purity as the sum of the different pectin-related monosaccharides (GalA, Gal, Ara, Rha, Xyl, and Fuc), all expressed in g per 100 g of the extracted material, and this purity is shown in Figure 4.

**Figure 5:**
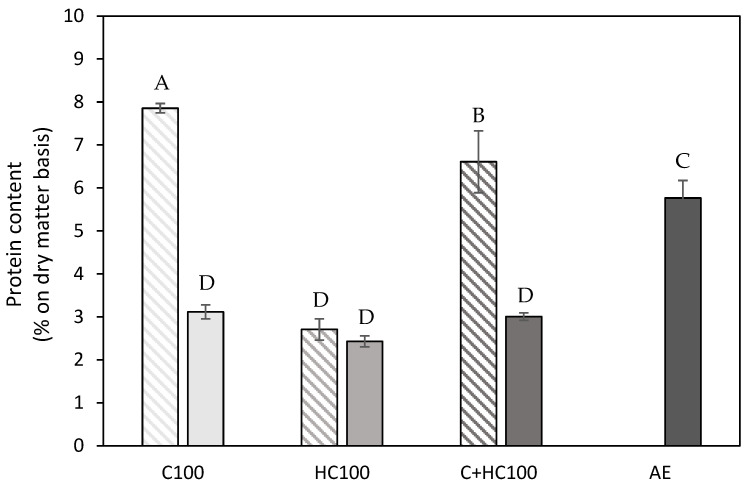
Protein content (% on dry matter basis) present in the extracts using the different enzyme-assisted extractions (cellulase (C), hemicellulase (HC), combination (C + HC)) without (shaded) and with (full) heat treatment of 5 min at 80 °C, in comparison to an acid extraction (AE) (dark grey). 100: concentration of 100 U/g for each enzyme. The error bars represent the standard deviation. A Tukey HSD test with *p* < 0.05 was used to evaluate whether differences in protein content were significant (different letters).

It can immediately be observed that the lowest pectin purity was achieved using hemicellulase (HC) without and with a heat treatment. These lower purities can be attributed to the higher hemicellulose co-extraction in these samples, as, in Table 1, a higher glucose and mannose content can be observed for these samples, which was also observed in the literature [14,20,21,26,43]. The co-extraction of cellulose, on the other hand, was negligible in these samples, as was expected. For the samples with the addition of cellulase (C), the co-extraction of both hemicellulose and cellulose was negligible (Table 1), as well as for the acid extracted (AE) sample, which explains the comparable pectin purity in these samples. This comparable glucose (and mannose) content between these samples (C100 and AE) was already reported in the literature for the EAE process from lime peel and sisal waste [19,44]. For the samples with the addition of both enzymes (C + HC), a slightly lower pectin purity was observed compared to the sample with only C, which can be attributed to the slightly higher extent of both hemicellulose and cellulose co-extraction. Additionally, it can be observed that the addition of a heat treatment resulted in a (significantly) increased pectin purity for all enzyme(s) (combinations). To find an explanation for these differences in pectin purity, the protein content of the different samples was determined as well (Figure 5). For the C100 and C + HC100 samples, the differences in purity can mainly be attributed to the difference in protein content, as the protein content in these samples significantly decreased after the heat treatment at 80 °C. 

The higher protein content in the C100 and C + HC100 samples can be explained by the fact that (some of) the enzymes ended up in the extracted materials, and the comparable protein content in both HC100 samples indicated that this was mainly the case for the cellulase. As the heat treatment may cause the enzymes to denature and aggregate, they might be retained by the filter during filtration, resulting in lower protein content in the extracted materials after the addition of heat treatment. For the HC100 samples, the difference in pectin purity without and with a heat treatment can mainly be attributed to differences in hemicellulose co-extraction. Compared to the different EAE samples with a heat treatment, a slightly higher protein content (5.5–6 vs. 2.5–3%) is observed in the AE sample, showing slightly more co-extraction of protein using an acid extraction. The protein contents reported for the different EAE processes were in line with previous findings for EAE from chicory roots in the work of Panouillé et al. (2006) [17]. For the AE sample, the pectin purity and protein content were in line with previous reports for an AE on carrot tissue in the literature [45]. For the different EAE samples, comparison with the literature data was not always feasible. On the one hand, because a structural characterization was often lacking, only relative monosaccharide concentrations were reported [44,46]. On the other hand, because the type of enzyme used was not specified, a combination of a cell wall degrading enzyme with a protease or pectinolytic enzyme was used [17,41].

#### 3.1.4. Molecular Structure of the Extracted Pectin

Finally, the molecular structure of the extracted pectins was studied in more detail by evaluating different monosaccharide ratios, the degree of methylesterification, and the weighted average molar mass (Table 2) of the different extracted materials. The different sugar ratios were calculated as described by Denman & Morris (2015) [39] with minor modifications. For the relative contributions of the HG and RG I domains to the pectin structure, in general, only minor differences can be observed between the EAE with cellulase (C) and the combination of both enzymes (C + HC) and the AE. A monosaccharide composition comparable to the AE was already reported in the work of Yang et al. (2018) [19] for an EAE with cellulase on sisal waste. In the ratio showing the branching of RG I, which is, in fact, a measure for the length of the RG I sides chains, slightly lower values were achieved for the AE sample compared to the EAE samples with cellulase (C) and the combination of both enzymes (C + HC). These slightly lower values for the AE sample can be attributed to the acid hydrolysis of the RG I side chains, mainly the ones consisting of arabinose, under the acidic extraction conditions (lower pH), as these side chains are more acid-sensitive [47]. This decrease in arabinose content with an AE was also reported in the work of Dominiak et al. (2014) [44] for the pectin extraction from lime peel. Interpretation of the results for the EAE with hemicellulase (HC) is more difficult, as these results are strongly influenced by the higher glucose and mannose content in these samples, resulting from the higher extent of hemicellulose co-extraction. In general, the addition of the heat treatment has a negligible impact on the sugar ratios of the different extracted materials.

In Table 2, which shows the degree of methylesterification (DM) for the different samples, it can be observed that all EAE treatments result in a comparable DM value, except for the slightly lower DM value in the HC100 sample. These comparable DM values using different cell wall degrading enzymes were also observed in the work of Idrovo Encalada et al. (2019) [16] on carrot tissue, although the DM values reported in the current study were higher. The order of magnitude for the DM values in this study was more in line with the results of Neckebroeck et al. (2020) [45] for an acid extraction on carrot tissue. When comparing the EAE processes with the AE, slightly higher DM values were found using the different EAE processes. Yang et al. (2018) [19] reported the same trend for the EAE of pectin using cellulase from sisal waste, as did Panouillé et al. (2006) [17] for chicory roots, without specifying the enzymes that were used, as well as Dominiak et al. (2014) [44], who used different cell wall degrading enzymes on lime peel.

Lastly, the weighted average molar mass was determined to gain more insight into the length of the extracted polymers. In Table 2, it can be observed that the AE resulted in a pectin polymer with a larger molar mass compared to different EAE samples. These lower molar masses obtained with the different EAE processes can probably be attributed to the slight pectinolytic side activity of the enzymes that were used. In the literature, it was described that these varying results for the molar mass are mainly caused by the pectinolytic activity of the cell wall degrading enzymes. For pumpkin tissue and chicory roots, lower molar masses were reported for the EAE processes [17,18,23], whereas the work of Wikiera et al. (2015) and (2016) [20,22] showed that an extraction process with the use of enzymes results in a higher molar mass if the enzymes have no or negligible pectinolytic activity. In the current study, it was confirmed spectrophotometrically that the cellulase and hemicellulase enzymes had a slight pectinolytic side activity, explaining the slightly lower molar masses obtained using the different EAE processes. Additionally, a slight but non-significant increase in molar mass can be observed for all EAE processes when the heat treatment is added, which might indicate that a heating step is needed to further liberate the pectin polymers of a (slightly) higher molar mass.

### 3.2. Combination of an Enzyme-Assisted Extraction with an Acid Extraction

In the second part of this research, the most promising enzyme-assisted extraction process was combined with an acid extraction, either as an additional extraction step on the acid residue or as enzymatic pretreatment before the acid extraction. Based on the results obtained in the previous part of this study, it was decided to use the EAE process with cellulase for these combination treatments. As the extraction process with cellulase and the combination of both enzymes resulted in a comparable pectin extraction yield and molecular structure, the extraction process with only cellulase was selected to avoid the additional cost and the slight hemicellulose and cellulose co-extraction when adding hemicellulose. In the experimental approach of this part of this study, both extraction steps of the combination treatments were completely separated to gain more insight into the amount of pectin and the pectin structure that was obtained in the different extraction steps.

**Figure 6:**
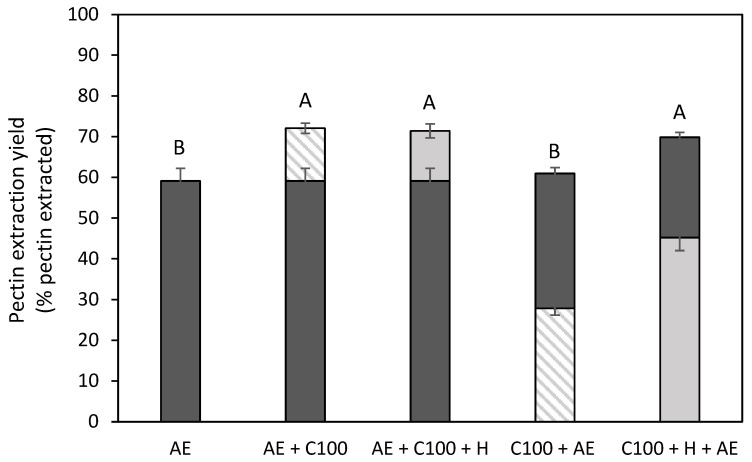
Pectin extraction yield (% pectin extracted) obtained after the combination treatments of the acid extraction and the enzyme-assisted extraction with cellulase (C) without (shaded) and with (full) heat treatment of 5 min at 80 °C, in comparison to an acid extraction (AE) (dark grey). Bottom bars: first extraction step, Top bars: second extraction step. 100: concentration of 100 U/g for each enzyme. The error bars represent the standard deviation. 95% confidence intervals were used to evaluate whether differences in total pectin extraction yield were significant (different letters).

Figure 6 shows the pectin extraction yield for the different combination treatments in comparison to the acid extraction. The bottom and top bars in this graph represent the first and second extraction steps, respectively. It can be observed that, besides the EAE treatment without a heat treatment (C100) followed by an AE (C100 + AE), all combination treatments resulted in a significantly increased pectin extraction yield (from 59 to 70–72%). Panouillé et al. (2006) [17] also studied the combination of the EAE (with cellulase and protease) with an acid extraction on chicory roots and also separated both extraction steps. Milošević & Antov (2022) [18], who studied an EAE with cellulase and xylanase combined with an acid extraction without the separation of the different extraction steps, also observed this increased extraction yield (on a mass basis) for pumpkin tissue. Also, for lemon peel, an increased extraction yield (on a mass basis) was observed for the combination treatment with an EAE process using xylanase [48]. When comparing both combination treatments with the EAE process with heat treatment (AE + C100 + H and C100 + H +AE), it can be observed that the order of the extraction steps has no influence on the total amount of pectin that was extracted, which was also observed in the study of Panouillé et al. (2006) [17] for chicory roots. The lower total pectin yield in the C100 + AE samples shows that the additional heat treatment after the EAE treatment is needed to obtain a comparable pectin extraction yield to the other combination treatments. When the biomass first underwent an extraction step at an elevated temperature, which was the case for the AE + C100 sample, this heat treatment did not lead to a further increased pectin extraction yield. When comparing the same extraction treatments performed in the first or second extraction step, it can be observed that all treatments always resulted in a lower pectin extraction yield when performed in the second extraction step because simply less pectin was present in the residue after the first extraction step.

To gain more insight into the composition of the extracted materials, the pectin purity and the protein content were determined (Table 3). Again, a distinction was made between the extracted materials obtained in the first and second extraction step of the combination treatments. Overall, the pectin purity varied from 68.4 to 79.5%, and it can be observed that the AE resulted in slightly higher pectin purity when performed after an enzymatic pretreatment. A possible explanation for these higher purities can be the fact the co-extraction of protein in these samples is lower because part of the protein was already co-extracted during the first enzymatic extraction step. For the enzymatic treatment, no clear influence of the preceding AE could be observed. Additionally, all enzymatic treatments without heat treatment (AE + C100—extraction step 2 and C100 + AE—extraction step 1) resulted in slightly lower pectin purity, which was already observed in the first part of this research, and these lower purities can be attributed to the greater extent of protein co-extraction in these samples, which was also described earlier.

Different sugar ratios, the degree of methylesterification, and the weighted average molar mass were again determined for the extracted pectins obtained in both extraction steps of the combination treatments (Table 4). In general, no pronounced differences in the relative contribution of HG and RG I to the pectin structure could be observed between the different extracts, except for the second extraction step in the C100 (+H) + AE treatments. For these samples, a significantly higher contribution of the RG I domain to the pectin structure could be observed. This indicates that the enzymatic pretreatment facilitates the extraction of pectin structures richer in RG I, which are normally more difficult to extract, during the subsequent acid extraction. When considering the branching of the RG I domain, which is, in fact, a measure for the length of the galactan and/or arabinan side chains, it can be observed that the lowest ratio is obtained for the enzymatic treatments performed on the acid residue (AE +C100 (+H)), whereas the highest ratio is observed for the EAE performed as first extraction step. As the lower branching ratio for the AE + C100 (+H) samples can mainly be attributed to the lower arabinose content in these samples), it can be suggested that the acidic conditions during the preceding AE resulted in the debranching of the RG I arabinan side chains, because these side chains are known to be more sensitive to acidic conditions [39,47]. Additionally, it was observed that performing the AE as the first extraction step or after the enzymatic pretreatment has no influence on the branching of the RG I domain.

For the DM, the pectins extracted in the second extraction step resulted in a slightly lower DM value for both combinations, which was also observed in the study of Panouillé et al. (2006) [17]. For the enzyme-assisted extraction performed on the acid residue, this decrease can potentially be attributed to the partial acidic demethoxylation of the pectin that remained in the residue after the acid extraction [49,50]. For the acid extraction on the enzymatic residue, the influence of the preceding enzymatic treatment was less outspoken. Liu et al. (2023) and Milošević & Antov (2022) [18,48] also reported a lower DM for the combination treatment (EAE + AE) compared to the AE. Although significant differences in DM were observed, they are anticipated to be of little practical relevance.

Finally, the average weighted molar mass was evaluated (Table 4). For the enzyme-assisted extraction, the preceding AE seemed to have a negligible influence, whereas the enzymatic pretreatment before the acid extraction resulted in the extraction of larger polymers. These larger polymers after the enzymatic pretreatment can possibly be attributed to the larger contribution of RG I in these samples, resulting in more voluminous particles and, therefore, an apparently larger molar mass. Analogous to the previous part of this research, it can be observed that the EAE processes result in a lower molar mass, which can potentially be attributed to the pectinolytic side activity in the enzymes. It should be noted that, for the second part of this study, comparison with the literature data was not always feasible, as the few studies in the literature on the combination of enzyme-assisted and acid extraction mostly did not make the distinction between the consecutive extraction steps, except for Panouillé et al. (2006) [17], who used an EAE process with both a cellulase and a protease on a completely different biomass, chicory roots. In the current study, the experimental approach with separation of the different extraction steps was selected to gain more insight into the contribution of both treatments on pectin yield and structure.

## 4. Conclusions

In the first part of this study, the effect of two cell wall degrading enzymes, cellulase (C) and hemicellulase (HC), and the combination of these two enzymes, during the enzyme-assisted extraction (EAE) of pectin from carrot pomace was studied. From this experimental setup, it could be concluded that the EAE with C and the combination of both enzymes, C + HC, resulted in the highest pectin extraction yield of all EAE processes. Additionally, the addition of HC (when using C) seemed to have a negligible impact on the pectin yield and only resulted in some co-extraction of cellulose and hemicellulose. Overall, the highest extraction yield was still achieved with acid extraction (AE); however, up to 80% of the pectin extraction yield with the acid extraction was achieved with the optimal EAE process despite the much milder extraction conditions (lower temperature and higher pH). In general, the C100 (+H) and C + HC100 (+H) samples resulted in a pectin structure comparable to the AE, but the extracted materials obtained with the EAE processes had a lower molar mass. Because of the minor differences between the C100 (+H) and C + HC100 (+H) samples, in the follow-up experiments, only the EAE process with cellulase was used.

In the second part of this research, the potential of the EAE with cellulase in combination with an acid extraction, as a pretreatment or as an additional treatment on the acid residue, was studied in more detail. Except for the combination C100 + AE, all combination treatments gave rise to a significantly increased pectin extraction yield, up to a total pectin extraction yield of 72%. However, as the additional yield achieved with the implementation of the EAE process (as pretreatment or as additional treatment) is quite limited, the additional cost of the use of the enzyme and the energy use should be considered to make reasoned decisions on the profitability of these extraction processes. Additionally, it should be noted that, despite the increase in the pectin extraction yield with these combination treatments (up to 72%), still approx. 28% of the pectin was left in the final residue, indicating that the extraction of all the pectin initially present seems not feasible. In general, the order of the different extraction steps (especially when combined with heat treatment) had a negligible influence on the total pectin extraction yield, but some differences in the molecular pectin structure could be observed. The major differences were the higher contribution of the RG I domain in the pectins extracted with the AE on the enzymatic residue and the less branched RG I domains in the materials extracted with an EAE on the acid residue. Also, different weighted-average molar mass values were reported for the different samples. All these results indicate that an additional amount of pectin with a slightly different structure can be extracted with the addition of the EAE process.

Overall, it can be concluded that the EAE with cellulase or a combination of both enzymes can potentially be used as a more environmentally friendly alternative for acid extraction and that the combination of the EAE with the acid extraction can be used to improve the extraction efficiency. The limited differences in pectin molecular structure (mainly DM and molar mass) are anticipated to have a minor impact on functional properties, which could be experimentally verified in future research, which is needed in order to move towards implementation into industrial pectin extraction processes.

## Figures and Tables

**Table 1:** Co-extraction of hemicellulose, indicated by the glucose and mannose content after hydrolysis with 4% H_2_SO_4_, and the co-extraction of cellulose, indicated by the additional glucose content after hydrolysis with 72% H_2_SO_4_, in the extracted materials obtained after the different enzyme-assisted extractions (cellulase (C), hemicellulase (HC), combination (C + HC)) without and with heat treatment of 5 min at 80 °C, in comparison to an acid extraction (AE). 100: concentration of 100 U/g for each enzyme, H: heat treatment, Glc: glucose, Man: mannose, n.d.: not detected.

g/100 g Extracted Material	Co-Extracted Hemicellulose		Co-Extracted Cellulose
	Glucose (Glc)	Mannose (Man)	Glucose (Glc)
	(4% H_2_SO_4_)		(72% H_2_SO_4_–4%% H_2_SO_4_)
C100	0.80 ± 0.11	0.64 ± 0.10	0.24 ± 0.34
C100 + H	1.08 ± 0.31	0.48 ± 0.20	n.d.
HC100	39.08 ± 4.44	5.34 ± 0.97	2.63 ± 10.04
HC100 + H	34.82 ± 1.93	4.47 ± 1.03	3.44 ± 6.73
C + HC100	5.68 ± 1.53	0.69 ± 0.21	7.28 ± 2.23
C + HC100 + H	6.12 ± 0.71	0.40 ± 0.10	6.51 ± 1.51
AE	0.96 ± 0.22	0.02 ± 0.01	n.d.

**Table 2:** The sugar ratios, degree of methylesterification (%), and average molar mass (kDa) of the extracts using the different enzyme-assisted extractions (cellulase (C), hemicellulase (HC), combination (C + HC)) without and with a heat treatment of 5 min at 80 °C, in comparison with an acid extraction (AE). 100: concentration of 100 U/g for each enzyme, H: heat treatment. A 95% confidence interval was used to evaluate whether differences in the different sugar ratios were significant (different letters), and a Tukey HSD test with *p* < 0.05 was used to evaluate whether differences in DM and weighted-average molar mass were significant (different letters). The error bars for the molar masses represent the standard deviation of the replicate measurements.

	Sugar Ratios			Degree of Methylesterification (%)	Weighted-Average Molar Mass (kDa)
	Contribution of HG (%) GalA-Rha	Contribution of RG I (%) 2Rha + Gal + Ara	Branching of RG I (-) (Ara + Gal)/Rha		
C100	72.72 ± 4.80 ^A^	24.88 ± 2.07 ^A^	5.27 ± 0.95 ^B^	68.89 ± 0.36 ^A^	108 ± 10.5 ^BCD^
C100 + H	68.36 ± 5.20 ^AB^	29.04 ± 4.38 ^A^	4.50 ± 1.33 ^BC^	66.80 ± 1.20 ^A^	168 ± 34.4 ^B^
HC100	25.34 ± 4.60 ^D^	12.72 ± 0.87 ^B^	44.4 ± 20.9 ^A^	61.83 ± 0.93 ^B^	88.6 ± 4.8 ^CD^
HC100 + H	33.84 ± 2.37 ^C^	11.47 ± 0.66 ^B^	46.3 ± 10.5 ^A^	67.38 ± 1.62 ^A^	154 ± 49.3 ^BC^
C + HC100	61.31 ± 1.99 ^B^	28.05 ± 3.86 ^A^	4.78 ± 1.16 ^B^	68.98 ± 0.62 ^A^	74.9 ± 16.2 ^D^
C + HC100 + H	67.55 ± 7.13 ^AB^	23.08 ± 3.25 ^A^	6.34 ± 2.23 ^B^	67.54 ± 0.94 ^A^	120 ± 24.1 ^BCD^
AE	73.27 ± 4.30 ^A^	25.12 ± 0.99 ^A^	3.10 ± 0.26 ^C^	62.94 ± 0.45 ^B^	282 ± 29.4 ^A^

**Table 3:** The pectin purity (%) and protein content (% on a dry matter basis) of the extracts using the different extraction steps of the combination treatments of the acid extraction and the enzyme-assisted extraction with cellulase (C) without and with a heat treatment of 5 min at 80 °C. A 95% confidence interval was used to evaluate whether differences in pectin purity were significant (different letters) and a Tukey HSD test with *p* < 0.05 was used to evaluate whether differences in protein content were significant (different letters).

		Pectin Purity (%)	Protein Content (% on Dry Matter Basis)
AE + C100	Extraction step 1—AE	73.97 ± 3.33 ^BC^	5.77 ± 0.41 ^B^
	Extraction step 2—C100	68.73 ± 3.76 ^C^	8.46 ± 0.08 ^A^
AE + C100 + H	Extraction step 1—AE	73.97 ± 3.33 ^BC^	5.77 ± 0.41 ^B^
	Extraction step 2—C100 + H	77.86 ± 4.37 ^AB^	4.04 ± 1.00 ^C^
C100 + AE	Extraction step 1—C100	68.38 ± 3.64 ^C^	7.86 ± 0.11 ^A^
	Extraction step 2—AE	76.69 ± 1.70 ^AB^	3.85 ± 0.09 ^C^
C100 + H + AE	Extraction step 1—C100 +H	75.89 ± 4.99 ^ABC^	3.12 ± 0.16 ^C^
	Extraction step 2—AE	79.49 ± 2.23 ^A^	3.61 ± 0.04 ^C^

**Table 4:** Sugar ratios, degree of methylesterification (%), and weighted-average molar mass (kDa) of the extracts using the different extraction steps of the combination treatments of the acid extraction and the enzyme-assisted extraction with cellulase (C) without and with heat treatment of 5 min at 80 °C. 100: concentration of 100 U/g for each enzyme, H: heat treatment. A 95% confidence interval was used to evaluate whether differences in the different sugar ratios were significant (different letters), and a Tukey HSD test with *p* < 0.05 was used to evaluate whether differences in DM and weighted-average molar mass were significant (different letters).

		Monosaccharide Ratios			Degree of Methylesterification (%)	Weighted-Average Molar Mass (kDa)
		Contribution of HG (%) GalA-Rha	Contribution of RG I (%) 2Rha + Gal + Ara	Branching of RG I (-) (Ara + Gal)/Rha		
AE + C100	Extraction step 1	73.27 ± 4.30 ^A^	25.12 ± 0.99 ^B^	3.10 ± 0.26 ^B^	62.94 ± 0.45 ^B^	282 ± 29.4 ^B^
	Extraction step 2	70.43 ± 5.13 ^A^	25.60 ± 0.91 ^B^	1.80 ± 0.13 ^C^	50.06 ± 2.22 ^E^	77.7 ± 0.72 ^C^
AE + C100 + H	Extraction step 1	73.27 ± 4.30 ^A^	25.12 ± 0.99 ^B^	3.10 ± 0.26 ^B^	62.94 ± 0.45 ^B^	282 ± 29.4 ^B^
	Extraction step 2	68.70 ± 5.07 ^A^	28.05 ± 2.43 ^B^	1.82 ± 0.32 ^C^	56.08 ± 1.24 ^D^	163 ± 53.8 ^C^
C100 + AE	Extraction step 1	72.72 ± 4.80 ^A^	24.88 ± 2.07 ^B^	5.27 ± 0.95 ^AB^	68.89 ± 0.36 ^A^	108 ± 10.5 ^C^
	Extraction step 2	61.17 ± 1.35 ^B^	37.90 ± 2.38 ^A^	3.24 ± 0.42 ^B^	56.95 ± 1.24 ^CD^	505 ± 59.3 ^A^
C100 + H + AE	Extraction step 1	68.36 ± 5.20 ^A^	29.04 ± 4.38 ^B^	4.51 ± 1.33 ^A^	66.80 ± 1.20 ^A^	168 ± 34.4 ^BC^
	Extraction step 2	58.48 ± 2.23 ^B^	40.63 ± 2.47 ^A^	3.40 ± 0.45 ^B^	59.22 ± 0.85 ^C^	580 ± 91.2 ^A^

## Data Availability

The original contributions presented in this study are included in the article and Supplementary Materials. Further inquiries can be directed at the corresponding author.

## Supplementary Materials

The following supporting information can be downloaded at https://www.mdpi.com/article/10.3390/foods14030435/s1. Table S1. Monosaccharide composition (g/100 g extracted material) of the extracted materials ob-tained after the different enzyme-assisted extractions (cellulase (C), hemicellulase (HC), combina-tion (C+HC)) without and with a heat treatment of 5 min at 80 °C, in comparison to an acid ex-traction (AE). Table S2. Monosaccharide composition (g/100 g extracted material) of the extracted materials obtained in the different extraction steps (S) of the combination treatments of the acid extraction and the enzyme-assisted extraction with cellulase (C) without and with a heat treatment of 5 min at 80 °C.
