# Supplementary material for: The Use of Different Cell Wall Degrading Enzymes for Pectin Extraction from Carrot Pomace, in Comparison to and in Combination with an Acid Extraction
Source: Foods. 2025 Jan 28;14(3):435. doi: 10.3390/foods14030435 (PMC11817013; doi:10.3390/foods14030435)
Supplement: Supplementary file 1 [file foods-14-00435-s001.zip › foods-3358614-supplementary.pdf]

Supplementary Materials

Table S1. Monosaccharide composition (g/100 g extracted material) of the extracted materials obtained after the different enzyme-assisted extractions (cellulase (C), hemicellulase (HC), combination (C+HC)) without and with a heat treatment of 5 min at 80 °C, in comparison to an acid extraction (AE). 100: concentration of 100 U/g for each enzyme, H: heat treatment. GalA: galacturonic acid, Gal: galactose, Ara: arabinose, Rha: rhamnose, Xyl: xylose, Fuc: fucose, Glu: glucose, Man: mannose, n.d.: not detected.

	**Monosaccharide content (g/100 g extracted material)**							
	Pectin-related						Co-extracted	
	GalA	Gal	Ara	Rha	Xyl	Fuc	Glu	Man
**C100**	55.0 ± 3.4	6.31 ± 0.91	4.82 ± 0.66	2.09 ± 0.32	0.09 ± 0.03	0.06 ± 0.01	0.80 ± 0.11	0.64 ± 0.09
**C100 + H**	58.9 ± 4.1	7.98 ± 2.21	5.92 ± 1.59	2.79 ± 0.68	0.21 ± 0.08	0.12 ± 0.04	1.08 ± 0.31	0.48 ± 0.20
**HC100**	19.9 ± 3.6	5.53 ± 0.45	2.71 ± 0.34	0.20 ± 0.08	0.22 ± 0.05	n.d.	39.08 ± 4.44	5.34 ± 0.97
**HC100 + H**	26.5 ± 1.8	4.95 ± 0.40	2.50 ± 0.21	0.17 ± 0.03	0.19 ± 0.06	n.d.	34.82 ± 1.93	4.47 ± 1.03
**C+HC100**	43.9 ± 1.3	7.04 ± 2.22	4.38 ± 0.31	2.15 ± 0.38	0.16 ± 0.13	0.05 ± 0.03	5.68 ± 1.53	0.69 ± 0.21
**C+HC100 + H**	55.2 ± 5.6	7.50 ± 1.66	4.41 ± 0.94	2.02 ± 0.58	0.17 ± 0.03	0.10 ± 0.03	6.12 ± 0.71	0.40 ± 0.10
**AE**	59.9 ± 3.3	6.62 ± 0.50	3.53 ± 0.13	3.77 ± 0.21	0.09 ± 0.01	0.05 ± 0.01	0.96 ± 0.22	0.02 ± 0.01

Table S2. Monosaccharide composition (g/100 g extracted material) of the extracted materials obtained in the different extraction steps (S) of the combination treatments of the acid extraction and the enzyme-assisted extraction with cellulase (C) without and with a heat treatment of 5 min at 80 °C. 100: concentration of 100 U/g for each enzyme, H: heat treatment. GalA: galacturonic acid, Gal: galactose, Ara: arabinose, Rha: rhamnose, Xyl: xylose, Fuc: fucose, Glu: glucose, Man: mannose, n.d.: not detected.

		**Monosaccharide content (g/100 g extracted material)**							
		Pectin-related						Co-extracted	
		GalA	Gal	Ara	Rha	Xyl	Fuc	Glu	Man
**AE + C100**	S1	59.9 ± 3.3	6.62 ± 0.50	3.53 ± 0.13	3.77 ± 0.21	0.09 ± 0.01	0.05 ± 0.01	0.96 ± 0.22	0.02 ± 0.01
	S2	56.2 ± 3.7	7.83 ± 0.42	0.31 ± 0.06	4.15 ± 0.20	0.22 ± 0.01	n.d.	1.69 ± 0.04	0.73 ± 0.03
**AE + C100 + H**	S1	59.9 ± 3.3	6.62 ± 0.50	3.53 ± 0.13	3.77 ± 0.21	0.09 ± 0.01	0.05 ± 0.01	0.96 ± 0.22	0.02 ± 0.01
	S2	62.7 ± 4.1	9.28 ± 1.26	0.79 ± 0.32	4.69 ± 0.60	0.30 ± 0.04	0.06 ± 0.03	1.53 ± 0.11	0.53 ± 0.07
**C100 + AE**	S1	55.0 ± 3.4	6.31 ± 0.91	4.82 ± 0.66	2.09 ± 0.32	0.09 ± 0.03	0.06 ± 0.01	0.80 ± 0.11	0.64 ± 0.09
	S2	54.5 ± 0.9	12.8 ± 1.30	3.77 ± 0.39	5.34 ± 0.50	0.22 ± 0.05	0.09 ± 0.02	0.31 ± 0.15	0.02 ± 0.01
**C100 + H + AE**	S1	58.9 ± 4.1	7.98 ± 2.21	5.92 ± 1.59	2.79 ± 0.68	0.21 ± 0.08	0.12 ± 0.04	1.08 ± 0.31	0.48 ± 0.20
	S2	54.6 ± 1.7	14.36 ± 1.14	4.40 ± 0.64	5.77 ± 0.60	0.26 ± 0.04	0.11 ± 0.04	0.21 ± 0.02	0.04 ± 0.02

Disclaimer/Publisher's Note: The statements, opinions and data contained in all publications are solely those of the individual author(s) and contributor(s) and not of MDPI and/or the editor(s). MDPI and/or the editor(s) disclaim responsibility for any injury to people or property resulting from any ideas, methods, instructions or products referred to in the content.
